# Comment: Admission neutrophil-to-lymphocyte ratio to predict mortality in burn patients: a meta-analysis

**DOI:** 10.1186/s40635-025-00734-y

**Published:** 2025-03-27

**Authors:** Nosaibah Razaqi, Rachana Mehta, Shubham Kumar, Ranjana Sah

**Affiliations:** 1 Afghanistan Center for Epidemiological Studies, Herat, Afghanistan; 2 Faculty of Medicine, Ghalib University, Herat, Afghanistan; 3 Clinical Microbiology, RDC, Manav Rachna International Institute of Research and Studies, Faridabad, Haryana 121004 India; 4 Center for Global Health Research, Saveetha Medical College and Hospital, Saveetha Institute of Medical and Technical Sciences, Saveetha University, Chennai, India; 5 Department of Paediatrics, Dr. D. Y. Patil Medical College Hospital and Research Centre, Dr. D. Y. Patil Vidyapeeth (Deemed-to-Be-University), Pimpri, Pune, 411018 Maharashtra India; 6 Department of Public Health Dentistry, Dr. D. Y. Patil Medical College Hospital and Research Centre, Dr. D. Y. Patil Vidyapeeth (Deemed-to-Be-University), Pimpri, Pune, 411018 Maharashtra India

## Dear Editor,

The recent publication by Awad et al. [1] (2024) titled “Admission neutrophil-to-lymphocyte ratio to predict mortality in burn patients: a meta-analysis” makes a valuable contribution to our understanding of the neutrophil-to-lymphocyte ratio (NLR) as a prognostic marker in burn patient management. The authors have meticulously demonstrated that elevated NLR values can significantly predict mortality in burn patients. However, we believe that the impact and clinical applicability of their findings could be further enhanced by addressing certain methodological aspects that could strengthen the study’s conclusions and their relevance to clinical practice.

First, the study’s reliance on a meta-analytic approach pooling data from multiple studies introduces the potential for variability in study quality to affect the overall findings. It is commendable that the authors used the NIH Quality Assessment Tool for their analysis; however, integrating a sensitivity analysis that stratifies studies based on their quality would provide deeper insight. Such an analysis would help determine whether the conclusions drawn are robust across both high and lower-quality studies or if the predictive value of NLR is disproportionately influenced by the higher-quality studies [2]. This approach would not only reinforce the reliability of NLR as a biomarker, but also highlight the need for standardization in future research methodologies.

Additionally, the application of the Grading of Recommendations Assessment, Development and Evaluation (GRADE) system could significantly clarify the certainty of the evidence presented [3, 4]. The GRADE approach assesses the quality of the body of evidence and the strength of recommendations by examining risk of bias, inconsistency, indirectness, imprecision, and publication bias. Employing this system could offer a nuanced understanding of the degree to which one can be confident that an elevated NLR consistently correlates with higher mortality in burn patients. It would also provide a framework for developing clinical guidelines and recommendations based on the presented evidence.

Moreover, the discussion of NLR as a prognostic tool would benefit greatly from a comparison with other established inflammatory markers used in burn care, such as C-reactive protein (CRP), procalcitonin, or specific cytokine profiles. A comparative analysis could situate NLR within the spectrum of available biomarkers and perhaps delineate scenarios where NLR might offer unique advantages or limitations. For instance, exploring whether NLR provides earlier indications of patient deterioration compared to CRP or if it is more reflective of specific inflammatory pathways would be invaluable. This approach would not only validate the utility of NLR, but also guide clinicians in choosing the most appropriate marker based on specific clinical situations.

Furthermore, incorporating a discussion on the operational implications of adopting NLR as a routine measure in burn units could drive its clinical adoption. Addressing practical aspects such as the cost-effectiveness of regular NLR measurements, the required changes in laboratory protocols, and the training needs of healthcare staff would make the findings more relevant and actionable for institutions considering the integration of NLR into their patient assessment protocols.

The study by Awad et al. marks a significant step forward in burn care management. By addressing these methodological and contextual enhancements, future research could solidify the role of NLR not just as a standalone marker but as a pivotal component of a multidimensional assessment strategy, enhancing predictive accuracy and ultimately improving patient outcomes in burn care.

## Data Availability

Not applicable.
